# A Review on Optoelectrokinetics-Based Manipulation and Fabrication of Micro/Nanomaterials

**DOI:** 10.3390/mi11010078

**Published:** 2020-01-10

**Authors:** Wenfeng Liang, Lianqing Liu, Junhai Wang, Xieliu Yang, Yuechao Wang, Wen Jung Li, Wenguang Yang

**Affiliations:** 1 School of Mechanical Engineering, Shenyang Jianzhu University, Shenyang 110168, China; liangwf@sjzu.edu.cn (W.L.); jhwang@sjzu.edu.cn (J.W.); yang.xieliu@sjzu.edu.cn (X.Y.); 2 State Key Laboratory of Robotics, Shenyang Institute of Automation, Chinese Academy of Sciences, Shenyang 110016, China; ycwang@sia.cn; 3 CAS-CityU Joint Laboratory on Robotics, City University of Hong Kong, Kowloon Tong, Hong Kong 999077, China; 4 Department of Mechanical Engineering, City University of Hong Kong, Kowloon Tong, Hong Kong 999077, China; 5 School of Electromechanical and Automotive Engineering, Yantai University, Yantai 264005, China; yangwenguang@ytu.edu.cn

**Keywords:** optoelectrokinetics, optically-induced dielectrophoresis, micro/nanomaterials, separation, fabrication

## Abstract

Optoelectrokinetics (OEK), a fusion of optics, electrokinetics, and microfluidics, has been demonstrated to offer a series of extraordinary advantages in the manipulation and fabrication of micro/nanomaterials, such as requiring no mask, programmability, flexibility, and rapidness. In this paper, we summarize a variety of differently structured OEK chips, followed by a discussion on how they are fabricated and the ways in which they work. We also review how three differently sized polystyrene beads can be separated simultaneously, how a variety of nanoparticles can be assembled, and how micro/nanomaterials can be fabricated into functional devices. Another focus of our paper is on mask-free fabrication and assembly of hydrogel-based micro/nanostructures and its possible applications in biological fields. We provide a summary of the current challenges facing the OEK technique and its future prospects at the end of this paper.

## 1. Introduction

Accurate manipulation and fabrication of micro/nanomaterials in liquid is fundamental for a range of applications such as micro/nanoelectronics [1,2], biosensors [3,4], biomedicine [5,6], biosensing [7,8] and energy harvesting [9,10]. Various attempts have been made to make that happen. For example, silver nanoparticles were integrated into the luminol system to enable more efficient electrochemiluminescence and thereby allow for ultrasensitive detection of cardiac troponin [11]. Combining graphene and traditional integrated circuits, a high-mobility, high-resolution, and broadband image digital sensor was developed to capture ultraviolet, visible, and infrared light [12]. Gold nanoparticles were assembled into micro/nanowires to fabricate a flexible pressure sensor that offers a detection limit up to 25 Pa [13]. This sensor is also sensitive to pulses in different regions of the human body, offering an approach to facilitating the development of wearable devices.

To address real-world needs for manipulation and fabrication of micro/nanomaterials, a number of micro-/nano-scaled methods have been presented. Typical examples include microfluidic [14,15], acoustic [16,17], electrokinetics [18,19], magnetic [20,21], optical [22,23], thermal [24,25], and atomic force microscope [26,27] approaches. An emerging topic on the manipulation and fabrication of micro/nanoparticles is about how to develop a novel mechanism that can complement what a single technique can offer. The long-term aim is to create valuable techniques that can help extend the applicability of micro/nanomaterials. Combining acoustic and magnetic fields, an approach that can aggregate nanoparticles in the presence of a magnetic field and move them towards the wall of a microchannel under the action of an external acoustic wave was proposed [28]. This approach bears significant potential for application in target drug delivery and biomedical surgery. There is also a technique called the acoustofluidic tweezers, which integrates acoustic waves into a microfluidic system and offers a label-free and high-throughput way to isolate 110-nm particles from a mixture of micro/nanoparticles with a purity as high as 99% [29]. By incorporating white light source from light-emitting diode into a microfluidic system, the absorbance of micro/nanoparticles at different diameters as well as the bit error rate can be determined, which allows easy and rapid measurement of micro/nanoparticle concentration [30]. In addition, a hybrid isomotive dielectrophoresis (DEP)-microfluidic technique was proposed for label-free separation of same-/differently-sized micro/nanoparticles based on their crossover-frequency features [31]. This technique also enables reliable and repeatable separation and dynamically adjusting the purity and yield of separated beads.

A new hybrid and novel manipulation technique called optically-induced dielectrophoresis (ODEP) or optoelectrokinetics (OEK) was proposed for programmable, contact-free, flexible, automatic, dynamic, and rapid manipulation of micro/nano-entities [32]. OEK has attracted much attention in micro/nanomanipulation fields since it was invented in 2005. This technique combines the merits of optical, electrokinetics, and microfluidic schemes, offering a more versatile approach to micro-/nano-scaled manipulation and fabrication over other competing lab-on-a-chip techniques. Optically-projected images are custom-designed on a computer using graphic animation software. These images are then transmitted by a commercial projector and focused onto the surface of the OEK chip, thereby triggering the photosensitive material and changing the distribution of the external AC bias potential. Most of the AC bias potential will shift to the liquid layer, which generates a non-uniform electric field around the illuminated area. Meanwhile, various electrokinetics forces are produced. These forces are further exerted onto the micro/nanoparticles, driving, directing, and delivering them towards the intended destination programmatically and digitally. This is how optical-electrokinetics-microfluidics integration works. Featuring the use of optically-projected images as virtual electrodes to directly manipulate micro/nanoparticles and without referring to metal-based electrodes, OEK has been widely used in the manipulation, separation, assembly, and fabrication of micro/nano-entities as well as extraction of their intrinsic properties [33,34,35,36,37,38], which has shown a highly promising new strategy in the development of micro/nanomanipulation and fabrication communities. More recently, Berkeley Lights Inc., the inventor of OEK, has commercialized this technology in bio-related fields, offering a unique approach to take the development of biomedical and bioengineering sciences to a new level. Furthermore, it also shows the high potential for the use of this technology in material manipulation and fabrication to move from lab-oriented research to real-world applications.

Unlike previous review studies that only discuss some aspects of OEK-based microfluidics [39,40,41], we present a comprehensive review of OEK-based manipulation and fabrication of micro/nanoparticles. First, we summarize differently structured OEK chips and discuss their respective working principles. Then, we describe OEK-based manipulation and fabrication of micro-/nano-scaled materials without using any masks. Next, we review the use of OEK chips for fabricating various hydrogel-based structures and functional micro/nanodevices. Finally, we explain current and future challenges facing the OEK technique and possible innovations on it.

## 2. Optoelectrokinetics (OEK) Chips and Their Working Principles

**Figure 1:**
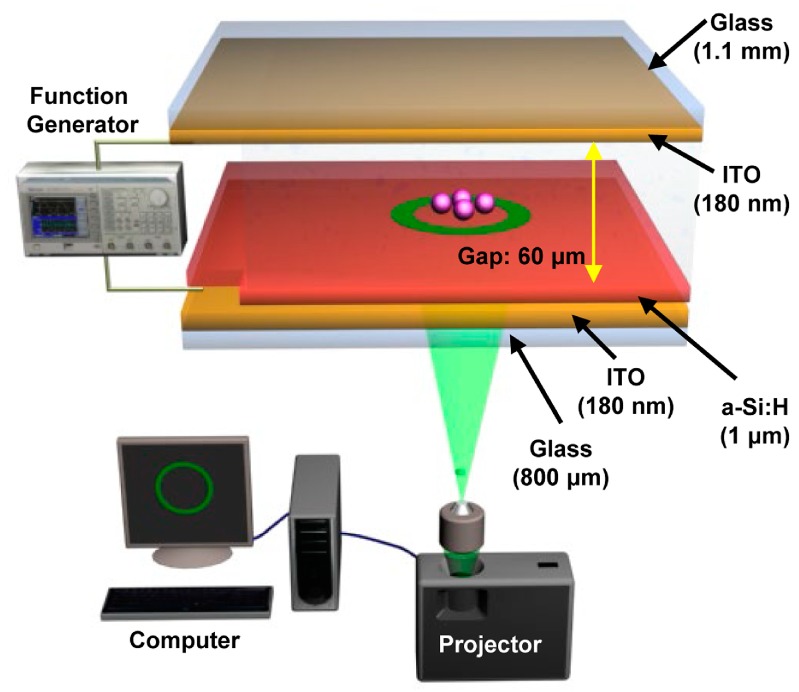
Schematic illustration of optoelectrokinetics (OEK) chip (reproduced from Ref. [42]).

The ODEP or OEK chip was firstly invented in 2005 [32]. The chip typically consists of four layers [32]: a top glass layer coated with a transparent and conductive indium tin oxide (ITO) film, which connects with one end of the AC bias potential; a bottom ITO glass layer serving as the bottom electrode that connects with the other end of the AC bias potential; a thin film (50 nm) n+ hydrogenated amorphous silicon (a-Si:H) layer, deposited onto the bottom ITO glass after generation of a 1 μm undoped a-Si:H layer and a 20 nm silicon nitride layer; a liquid layer that contains the manipulated micro/nanoparticles to function as a “gap” for the OEK chip. The doped a-Si:H layer can decrease the contact resistance between the liquid layer and the undoped a-Si:H layer, and the silicon nitride layer serves the purpose of passivation. It is worth noting that the undoped a-Si:H layer is the photosensitive layer from which optically-induced virtual electrodes are generated. In general, the ITO layer is sputtered onto the glass layer; the a-Si:H layer is deposited onto the ITO layer by plasma-enhanced chemical vapor deposition. Figure 1 is an illustration of its mechanism proposed in our group given in [42]. In our study, only a layer of a-Si:H was deposited, which was also classified into an a-Si:H based OEK chip.

When the OEK chip is illuminated by the optically-projected patterns shown on a digital micromirror display (DMD), the external AC voltage shifts to the liquid layer with suspended micro/nanoparticles. Then, an optically-induced non-uniform electric field is produced around the illuminated area. Under the action of this electric field, the micro/nanoparticles are polarized, producing an ODEP force. This force is, in turn, exerted onto the micro/nanoparticles, which is defined as [43]

(1) 〈F→ODEP〉=2πR3εmRe[K(ω)]∇|E→rms|2,

where *R* is the radius of the micro/nanoparticles, *ε* is the permittivity of the liquid, *E* is the magnitude of the optically-induced non-uniform electric field, *rms* means the root-mean-square value, the subscript *m* is the liquid, *ω* is the angular frequency of the AC bias potential across the liquid medium, and *K*(*ω*) is the Clausius–Mossotti (CM) factor, which is further expressed as

(2) $$K\left(\omega \right)=\frac{\varepsilon_{p}^{*}-\varepsilon_{m}^{*}}{\varepsilon_{p}^{*}+2\varepsilon_{m}^{*}},$$

where ε^*^ = *ε* − j*σ*/*ω*, with *σ* representing the conductivity. The subscript *p* means the particle. The direction of the ODEP force, both positive and negative, is fully directed by the real part of the CM factor. Specifically, if the value of the real part of the CM factor is higher than zero, a positive ODEP force will be generated and then the micro/nanoparticles will be attracted and moved to the illuminated area. Otherwise, the micro/nanoparticles will be pushed away from the illuminated area. For micro/nanoparticles that vary significantly in their size, a common method to separate them is by making the ODEP magnitudes have three orders of difference.

Other OEK forces, including AC electroosmosis (ACEO) and AC electrothermal, were investigated using the finite element analysis (FEA) method [44]. Typically, those two kinds of electrokinetics forces arise from the interaction between the optically-induced non-uniform electric field and liquid solution at a given AC frequency. As an example, under the action of an ACEO force, the fluid flow can rapidly push a large number of micro/nanoparticles towards the illuminated area and assemble them into desired patterns or devices [45,46].

**Figure 2:**
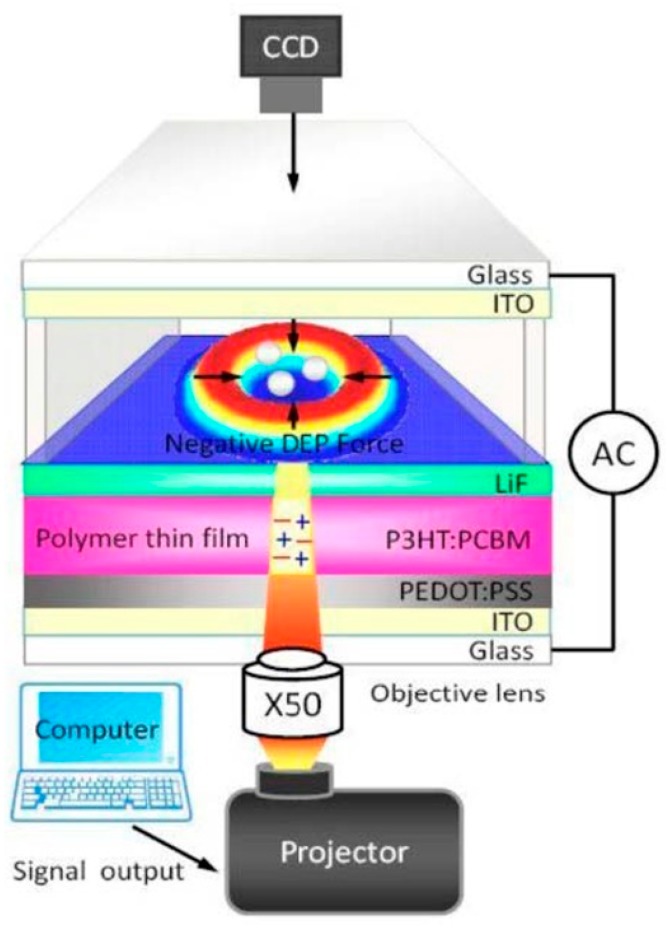
Structure of P3HT/PCBM polymer-based OEK chip (reproduced from Ref. [47]).

Recently, various types of photosensitive materials have been explored to manipulate and fabricate micro/nanomaterials in the same way as an a-Si:H based OEK chip works. A P3HT/PCBM bulk-heterojunction polymer was demonstrated as an alternative to a-Si:H for micro/nanomanipulation [47]. Figure 2 shows the structure of an OEK chip made up of this material. P3HT/PCBM serves as a photosensitive material to respond to optically-projected images and produce a non-uniform electric field, thereby manipulating and isolating differently sized polystyrene beads [48]. The P3HT/PCBM polymer for making this type of chip is created by spinning-coating at a relatively low temperature. Compared to a-Si:H based OEK chips, OEK chips based on this material are much easier to fabricate, except that the fabrication process of the latter requires preventing the polymer film from being collapsed by either water or oxygen. OEK chips based on this material can bend up and down freely to produce concave or convex curvatures, which leads to significantly higher efficiency in separating and concentrating differently sized polystyrene beads [49].

**Figure 3:**
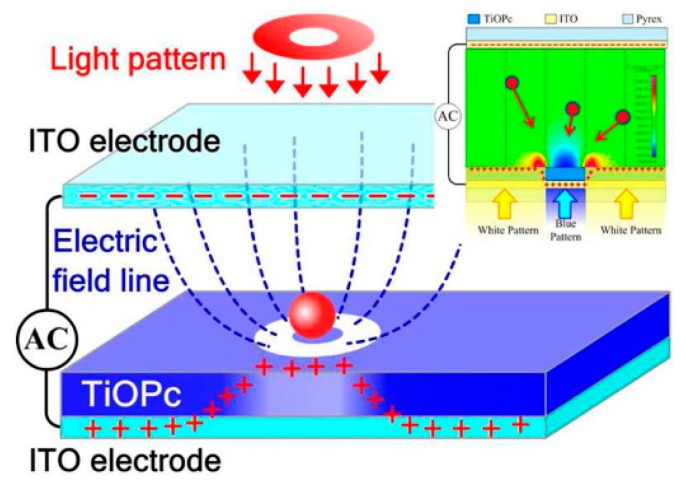
Structure of T_i_OP_c_-based OEK chip and simulation of the electric field (reproduced from Ref. [50]).

Another kind of OEK chip was devised based on T_i_OP_c_, an organic photosensitive material. As shown in Figure 3 [50], the chip has the same structure as the one indicated in Figure 1. This chip can be easily fabricated only by spinning-coating and baking techniques. This chip is fabricated in a much easier way than an a-Si:H based chip. It has been demonstrated that this chip can perform real-time manipulation of picobubbles [51] and droplets [52] as well as cell patterning [53]. However, problems remain with the long-term stability of this OEK chip.

## 3. OEK-Based Manipulation of Micro/Nanomaterials

### 3.1. Separation and Assembly of Micro-Scaled Particles

The OEK chip has been commonly used to perform manipulations such as separation, concentration and assembly on micro/nanoparticles, with the aid of an ODEP and/or ACEO mechanism. The OEK chip permits the assembly of 2D colloidal microparticles by using the electrohydrodynamic flows [54,55,56] and the manipulation of droplets [57,58,59]. Differently sized polystyrene beads serve various functions in material science and biomedical research. Hence, finding a rapid and automatic method to manipulate and separate them is critical in investigating the performance of targets of interest.

The conductivity of polystyrene beads is size-dependent and expressed as *σ* = 2*K_S_*/*R*, where *K_S_* is the surface conductivity of polystyrene beads [42]. The relationship between the crossover frequency (Re [*K*(*ω*)] = 0) of the ODEP force and the size of the polystyrene beads can be further derived as [42]

(3) $$f_{crossover}=\frac{1}{2\pi }\sqrt{\frac{\left(\sigma_{m}R-2K_{S}\right)\left(2K_{S}+2\sigma_{m}R\right)}{R^{2}\left(\varepsilon_{p}-\varepsilon_{m}\right)\left(\varepsilon_{p}+2\varepsilon_{m}\right)}}.$$

**Figure 4:**
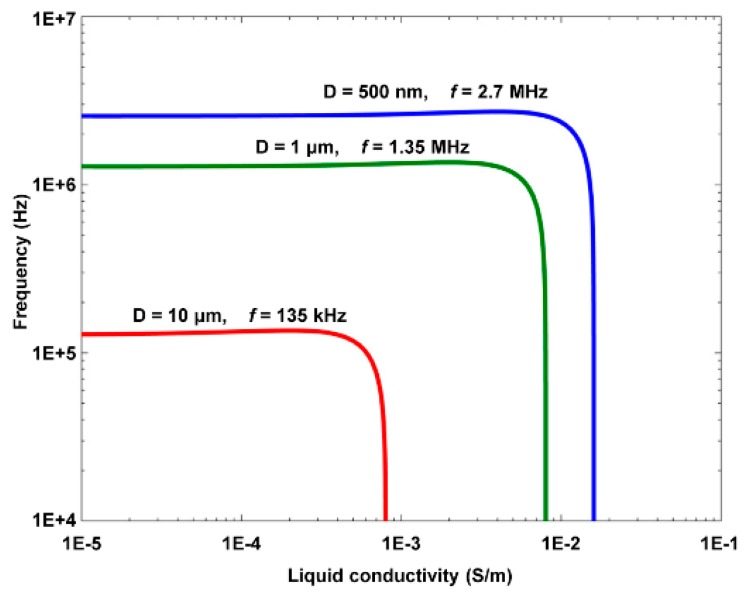
Crossover frequency vs. liquid conductivity of three differently sized polystyrene beads (reproduced from Ref. [42]).

**Figure 5:**
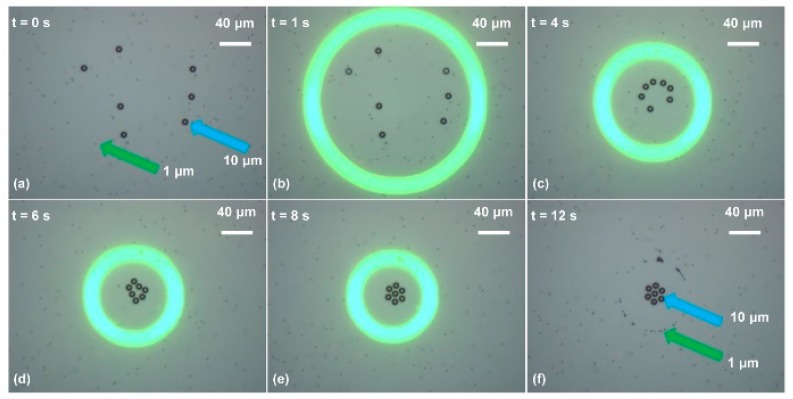
Separation of 10 μm and 1 μm polystyrene beads. (**a**) Initially, polystyrene beads were suspended in the liquid solution; (**b**) the OEK chip was illuminated by the optical ring pattern, with the AC bias potential switched on simultaneously; (**c**) 10 μm polystyrene beads were pushed towards the central area of the ring under the action of a negative ODEP force, while the 1 μm ones were attracted into the ring under the action of a positive ODEP force as the ring size decreased; (**d**,**e**) are captured images showing the positions of the polystyrene beads with two different diameters as the ring size decreased dynamically; (**f**) shows the final positions of the 10 μm and 1 μm polystyrene beads (reproduced from Ref. [42]).

The size-dependent crossover frequency as a function of the liquid conductivity, i.e., Equation (3), is illustrated in Figure 4 [42]. Then, separation of 1 μm and 10 μm polystyrene beads was successfully demonstrated by using ODEP forces in different directions, i.e., a positive ODEP force exerted onto the 1 μm polystyrene beads and a negative one onto the 10 μm ones, as shown in Figure 5 [42].

**Figure 6:**
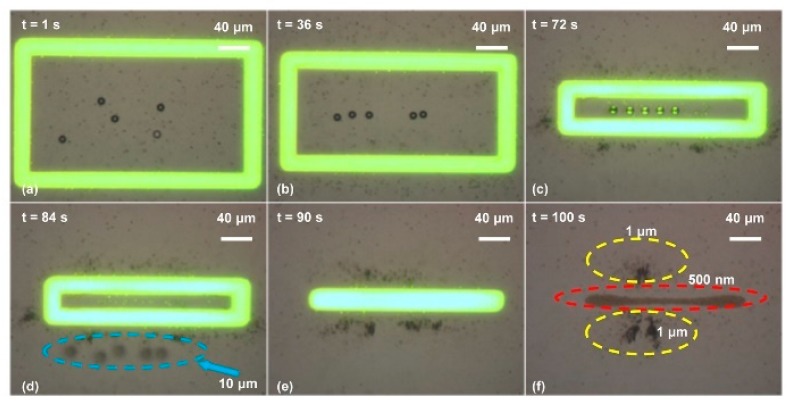
Simultaneous separation of polystyrene beads with diameters of 500 nm, 1 μm, and 10 μm. (**a**) When the OEK chip was illuminated by the optical rectangle pattern under the action of an external AC bias potential, both the 500 nm and 1 μm polystyrene beads were attracted to the illuminated area. By contrast, the 10 μm ones were pushed away from the optical ring; (**b**) as the optical rectangle size decreased, the 10 μm polystyrene beads moved towards the central area of the optical rectangle and those with the other two diameters still resided within the optical ring; (**c**) when the gap formed by the long sides of the rectangle was around 10 μm, the 10 μm polystyrene beads were aligned; (**d**) the 10 μm polystyrene beads were separated as the rectangle size further decreased; (**e**) the rectangle was decreased to a line pattern; (**f**) the 500 nm and 1 μm polystyrene beads were finally located at different positions of the OEK chip (reproduced from Ref. [42]).

Moreover, polystyrene beads with three different diameters, i.e., 500 nm, 1 μm, and 10 μm, were separated simultaneously. Figure 6 shows the detailed experimental process [42]. A negative ODEP force would be exerted onto the 10 μm diameter polystyrene beads, while a positive ODEP force would be exerted onto both the 500 nm and 1 μm ones. However, the 1 μm polystyrene beads would experience a much higher magnitude of ODEP force than the 500 nm ones because the magnitude of the ODEP force is proportional to the third power of the particle radius.

**Figure 7:**
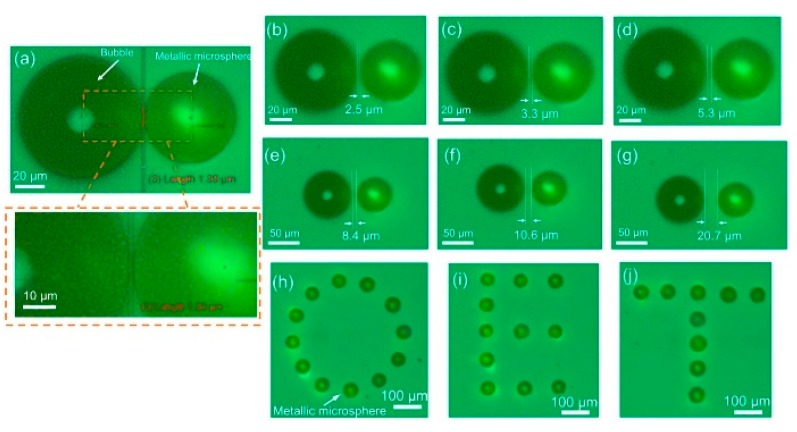
Captured images of manipulated metallic microparticles with different spaces to a bubble: (**a**) 1.39 μm, (**b**) 2.5 μm, (**c**) 3.3 μm, (**d**) 5.3 μm, (**e**) 8.4 μm, (**f**) 10.6 μm, and (**g**) 20.7 μm; parallel assembly of metallic microspheres into images of (**h**) “O,” (**i**) “E,” (**j**) “T” (reproduced from Ref. [60]).

Manipulation and assembly of metallic microspheres into patterns were proposed [60]. It was observed that conductive silver-coated poly(methyl methacrylate) (PMMA) microspheres (50 μm diameter) could move in an OEK chip at a maximum velocity of 3200 μm/s, much quicker than non-conductive microparticles such as polystyrene beads [61]. As the motorized XY stage moves at an increasing velocity, the microspheres reach their maximum velocity when they cannot follow the movement of the stage. Simulation on the electric field distribution and the ODEP force attributes the strong ODEP force to the local interaction between the optically-induced electric field and the silver shells surrounding the microspheres. The microspheres were then experimentally assembled to validate their high-accuracy positioning capabilities. Figure 7 shows how the microspheres were assembled with different spaces, i.e., from 1.39 μm to 20.7 μm, relative to a stable bubble [60]. The assembly of the “O,” “E,” “T” pattern was realized, demonstrating the ability of the OEK chip to precisely and parallelly pattern metallic microspheres into arbitrary shapes. In addition, the manipulation and assembly of hybrid metal-polymer microparticles were achieved [62].

**Figure 8:**
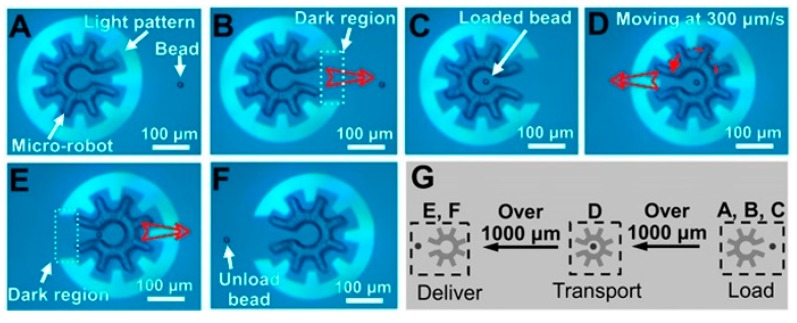
A series of OEK-based robotic micromanipulations. (**A**) A fully enclosed microrobot; (**B**) the load mode of a partially enclosed microrobot; (**C**) the partially enclosed microrobot loaded one bead of interest; (**D**) the fully enclosed microrobot transported the bead; (**E**) the partially enclosed microrobot delivered the bead; (**F**) the bead was unloaded; (**G**) schematic illustration of the “load,” “transport,” and “deliver” micromanipulations (reproduced from Ref. [63]).

It has been further demonstrated that OEK can serve as a versatile and programmable microrobot to perform typical micromanipulations such as “load,” “transport,” and “deliver” [63]. Firstly, OEK was used to manipulate custom-designed microstructures. Then, the OEK based microrobot could manipulate secondary microparticles in a parallel, multistep, contact-free and programmable manner. Figure 8 is a series of images showing the dynamic process of the OEK-based microrobot across large distances. This partially enclosed microrobot could load one 15 μm diameter polystyrene bead of interest with the aid of a negative ODEP force (Figure 8A–C), followed by a translational movement of 300 μm/s (Figure 8D). In addition, the OEK-based microrobot delivered this bead to the target location (Figure 8E,F). Figure 8G schematically presents the three-step process, which exhibited a higher moving velocity than when OEK was used alone. This OEK-based microrobot was also validated to be suitable for isolating single cells for clonal expansion and other biomedical applications.

### 3.2. Manipulation of Nano-Scaled Particles

OEK has also been used to dynamically manipulate nano-scaled entities, including the separation of nanowires [64,65,66,67], the patterning of two-dimensional nanomaterials [68], and manipulation of nanoparticles [69,70,71,72,73,74].

OEK allows the use of ODEP forces to effectively separate individual nanowires with different conductance levels, which has facilitated the development of nanodevices. Dynamic separation of semiconducting and metallic nanowires based on their difference in translational velocity was reported [64]. When the external voltage was higher than the “separation voltage,” i.e., the threshold one for moving the silicon nanowire, the silver nanowire could be separated from the silicon nanowire. Due to the high polarization of metallic nanowires in a non-uniform electric field, the silver nanowire moved at a much higher velocity than the silicon nanowire. When the OEK chip was dynamically illuminated by an optically-projected laser line at a scanning velocity greater than 2 μm/s and an AC bias potential of 8 V_pp_, both of these two types of nanowires were trapped by the laser line initially. The silicon nanowire, however, could not follow the movement of the laser line and became “uncontrollable.” Hence, these two types of nanowires were successfully separated. In addition, it was experimentally validated that real-time and large-scale assembly of silver nanowires is possible using an array of image-defined traps, suggesting the potential for massively parallel assembly at the nanoscale.

**Figure 9:**
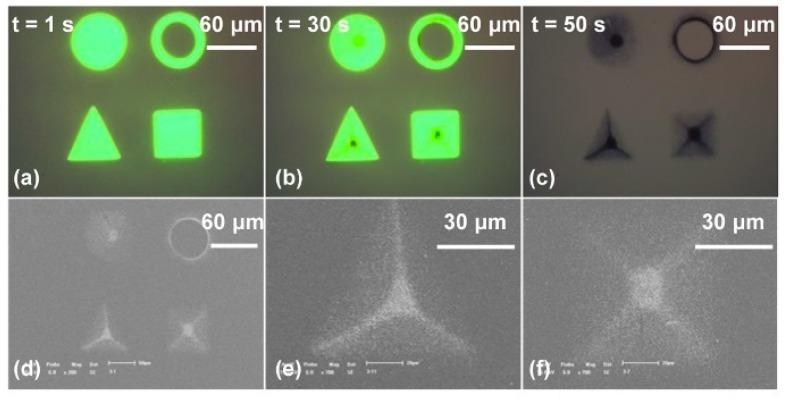
Rapid assembly of various microstructures using gold nanoparticles (AuNPs) with a diameter of 100 nm. (**a**) Four different optical patterns served as virtual electrodes, with an external AC bias potential switched on simultaneously; (**b**) after 30 s, the AuNPs were attracted into the areas illuminated by the four patterns; (**c**) AuNP-based microstructures formed when the optical patterns were moved and AC bias potential was switched off; (**d**) SEM images of (**c**); (**e**) SEM image of an AuNP-based microstructure in triangle pattern; (**f**) SEM image of an AuNP-based microstructure in square pattern (reproduced from Ref. [72]).

Furthermore, the manipulation and assembly of nanoparticles have been studied both theoretically and experimentally. First, a rapid and automatic assembly of 100 nm diameter gold nanoparticle (AuNP)-based microstructures was experimentally investigated [72]. Figure 9 indicates the experimental process in which AuNP-based microstructures were rapidly assembled. It was observed that each geometry of the four AuNP-based microstructures could reflect the optical pattern. However, using the triangle pattern as the virtual electrodes attracted most of the AuNPs into the locations of angular bisectors and formed a circle structure with three lines pointing toward the center of the triangle. The square pattern pushed the AuNPs to the diagonals of the pattern and most of them were moved into the central area.

Fabrication of various nanomaterial-based microelectrodes in an OEK chip was achieved using two different methods [73]. A composite solution consisting of conductive polyaniline (PANI) nanoparticles and multi-walled carbon nanotubes (MWCNTs) was used to examine the possibility of creating microelectrodes by OEK forces. Microelectrodes with various geometrical sizes could be fabricated within 1–3 min. Compared to the resistance change of the MWCNTs bundles, that of the microelectrodes could be neglected; the ethanol concentration was successfully reflected by the resistance change of the sensitive elements.

**Figure 10:**
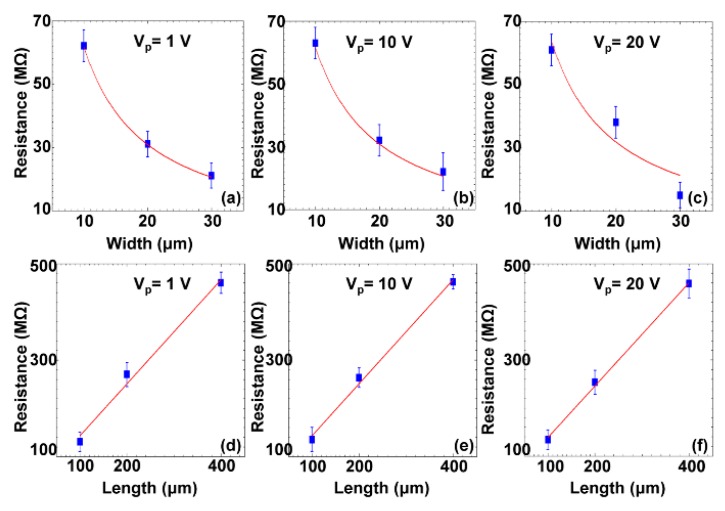
The curve-fitting function of the assembled carbon nanoparticle (CNP)-based microstructures. Resistance with respect to different widths of three microstructures at three different measurement voltages (**a**–**c**); Resistance with respect to different lengths of three microstructures at three different measurement voltages (**d**–**f**) (reproduced from Ref. [74]).

Rapid assembly of carbon nanoparticles (CNPs) with a diameter of 50 nm into electrical elements was realized [74]. A series of experiments were conducted to rapidly assemble CNPs into various electrical elements within 45 s. The results demonstrated that the microstructures had resistance properties. Specifically, their resistance value could be controlled by the width and length of the microstructures: inversely increasing with the width (Figure 10a–c) and linearly increasing with the length (Figure 10d–f).

## 4. Mask-Free Fabrication of Electrodes and Devices

An optically-induced electrochemical reaction and deposition scheme was presented to enable dynamic, rapid and mask-free fabrication of microelectrodes [75,76,77,78,79]. When the OEK chip was illuminated by optically-projected images, an electrical field would be produced in the illuminated area due to the creation of electron-hole pairs. Then, only the metal ions of liquid solution in the illuminated area were reduced by trapping electron from a-Si:H when an external AC bias potential was switched on. Furthermore, the reduced metal atoms would be attached to the illuminated a-Si:H surface and formed into metallic microstructures with the same shape as the incident light.

**Figure 11:**
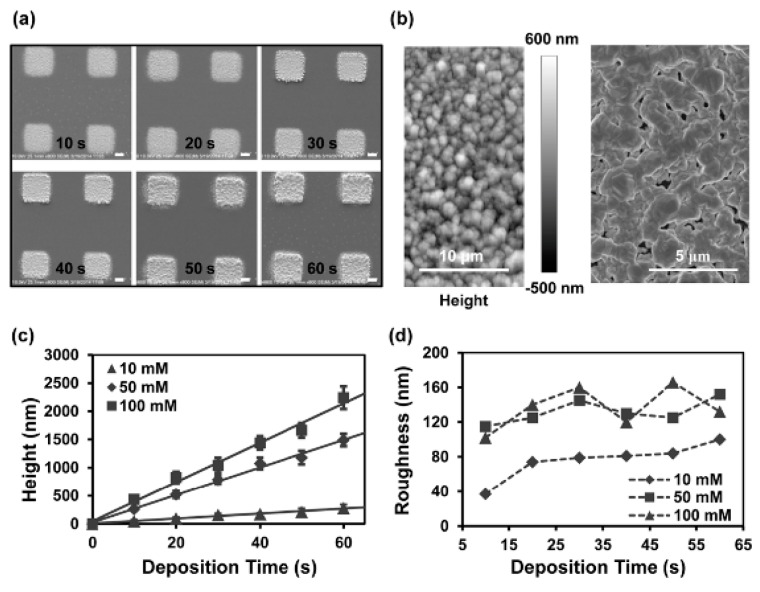
(**a**) Fabricated microelectrodes with respect to deposition time; scale bar: 10 μm. (**b**) Surface roughness of microelectrode by atomic force microscope (AFM) and SEM. (**c**) Thickness and (**d**) surface roughness of microelectrodes with respect to deposition time and solution concentration (reproduced from Ref. [75]).

Compared to the OEK-force-based method, this scheme could dynamically fabricate microelectrodes in 10 secs with a liquid conductivity as high as 2 × 10^7^ S/m. For instance, silver ion-based microelectrodes were reported, as shown in Figure 11 [75]. These microelectrodes exhibited more even distribution and lower roughness. Their heights could be adjusted by the linearly increasing deposition time and the solution concentration. In addition, the entire experiment was conducted at room temperature and atmospheric pressure without using conventional photolithographic techniques and metal nanoparticles and/or inks. In addition, integrated CuO/ZnO-based/single-walled-nanotube [76] nanowire-based field-effect transistors were presented, further validating that this scheme could facilitate the manufacturing of integrated nanodevices and offer an alternative to micro/nanosensor fabrication.

**Figure 12:**
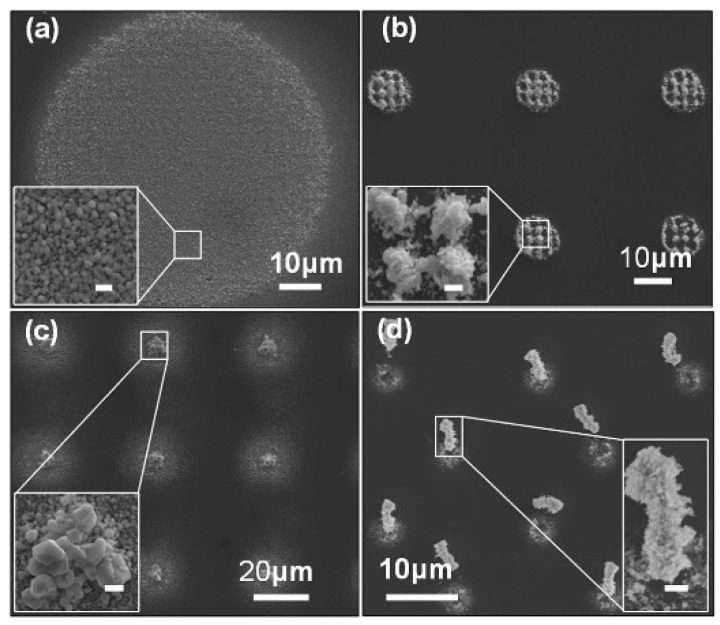
Synthesis of various silver nanostructures by optically-projected patterns in OEK chip. (**a**) Silver crystal nanoparticles. (**b**) Stacked silver hexagonal nanoplates. (**c**) Crystallized silver octahedra and hexagonal silver nanoplates. (**d**) An array of silver nanoparticles-based micropillars (reproduced from Ref. [77]).

Silver-based nanostructures with various geometrical topographies were synthesized in an OEK chip, as shown in Figure 12 [77]. The incident light excited the generation of electron-hole pairs in the a-Si:H layer and then the electrons migrated from the valence band to the conduction band. With the aid of an existing electric double layer, an electrochemical reaction occurred between the electrons and the suspended silver solution in the liquid chamber under given parameters in the solution; meanwhile, crystallization occurred with the silver-based nanostructures. In this case, silver polyhedral nanoparticles, nanoplates in triangle and hexagon patterns, as well as nanobelts were fabricated by projecting various optical patterns. Experimental parameters that affected the fabrication process, including time duration, AC frequency and voltage, were also investigated and optimized. In sum, this OEK-based method opens up a new path to mask-free and rapid synthesis of nanostructures and nanobelts.

**Figure 13:**
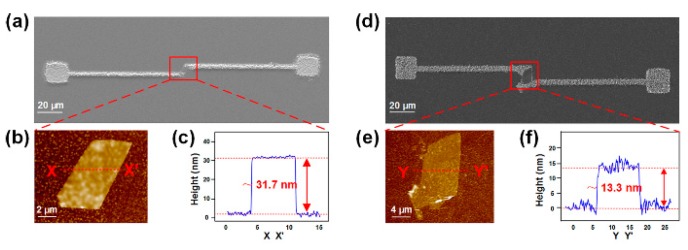
SEM images of MoS_2_ thin-film transistors fabricated with Au (**a**) and Ag (**d**) electrodes; (**b**,**e**) are the enlarged view of the rectangle areas in (**a**,**d**), respectively; (**c**,**f**) are the AFM characterization results of the heights of MoS_2_ thin-film transistors (reproduced from Ref. [78]).

Using this method, MoS_2_ with excellent optical and electronic properties was fabricated into thin-film transistors without relying on any conventional microlithography, such as nanoimprint lithography, laser patterning, or photolithography [78]. The MoS_2_ material was first loaded into the OEK chip. Then, the Au and Ag electrodes were rapidly fabricated onto the target MoS_2_ film. Accordingly, MoS_2_ thin-film transistors with Au and Ag electrodes were obtained, respectively, as shown in Figure 13. These transistors performed the best when they were 30–40 nm thick, exhibiting a low subthreshold swing of 0.75 V/decade and high mobility of 41 cm^2^·V^−1^·s^−1^, much better than conventional Si-based thin-film transistors.

## 5. Fabrication and Assembly of Hydrogel-based Micro/Nanostructures

Mask-free and non-UV based polymerization and prototyping of high-aspect-ratio 3D hydrogel microstructures, such as poly (ethylene glycol) (PEG)-diacrylate (PEGDA), was demonstrated in an OEK chip [80,81,82,83,84,85].

**Figure 14:**
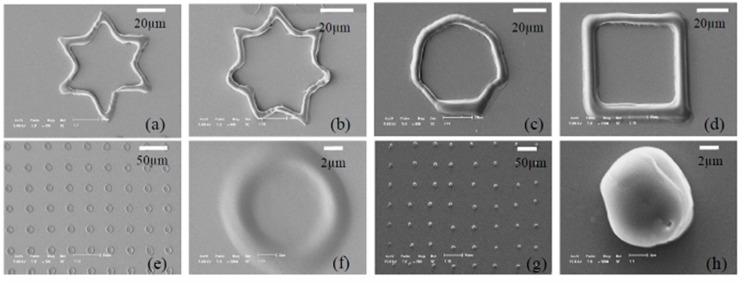
SEM images of poly (ethylene glycol) (PEG)-diacrylate (PEGDA)-based micro/nanostructures fabricated using optically-projected spot patterns with different exposure times. The heights in (**a**–**f**) measured by AFM were 233 nm, 455 nm, 687 nm, 950 nm, 1.34 μm, and 1.62 μm, respectively (reproduced from Ref. [80]).

A laser or UV-based method was proposed to obtain PEGDA-based micro/nanostructures [86,87]. The abovementioned OEK-based mask-free method could meet the same purpose without using any UV light sources or lasers [80]. Figure 14 shows the experimental results with controlled sizes, shapes and thicknesses of PEGDA-based structures. Parallel micro/nanostructures were flexibly patterned onto the chip using optical images. Furthermore, by projecting a series of custom-designed and dynamic optical patterns that served as digital masks, 3D microstructures were fabricated rapidly in a layer-by-layer manner, with their thickness varying from tens of nanometers to hundreds of micrometers [81].

**Figure 15:**
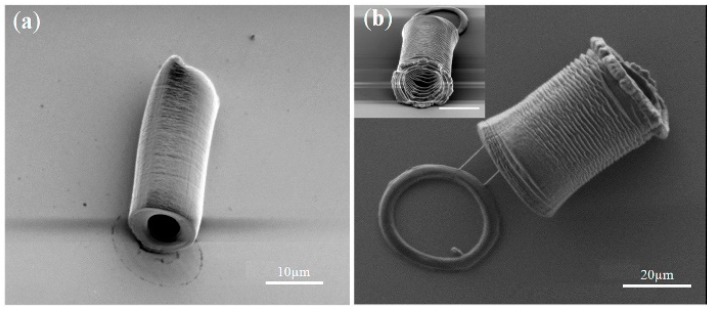
(**a**) PEGDA-based hydrogel tube with a length of ~20 μm, an outer diameter of ~10 μm, and an inner diameter of ~5 μm. (**b**) PEGDA-based hydrogel tube with a length of ~35 μm, an outer diameter of ~25 μm, and an inner diameter of ~20 μm (reproduced from Ref. [82]).

In addition, hollow and circular tubes were fabricated with a length, diameter, wall thickness, and high-aspect-ratio tuned by the exposure time, as shown in Figure 15 [82]. These tubes could manipulate and trap polystyrene beads when they grew longer as the exposure time increased. Then, the PEGDA-based tube continued to elongate and the beads were trapped and moved away from the initial position. Arrays of hydrogel micropillars were also fabricated. These micropillars were capable of serving as micro-scaled cavity molds for casting polydimethylsiloxane, demonstrating potential of finding applications in microfluidics-related fields. The abovementioned results indicate this technology is well-suited for the rapid fabrication of microfluidic chips.

**Figure 16:**
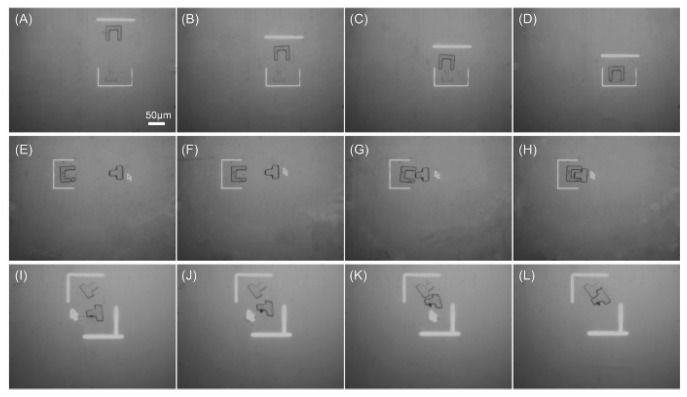
(**A**–**G**) Captured microscope image showing the dynamic process of assembling microstructures with different sizes and shapes; (**H**–**L**) the experimental process of assembling two different microstructures (reproduced from Ref. [83]).

Assembly of PEGDA-based microstructures was also realized, followed by an application of bottom-up functional tissue construction and engineering [83]. Arbitrary PEGDA-based microstructures were polymerized in a high-throughput and on-demand manner by a DMD. Then, the fabricated microstructures were transferred into an OEK chip for assembly, which was achieved by using microfluidic flow. Figure 16 illustrates the assembly of PEGDA-based microstructures with different sizes and shapes. Before moving into the OEK chip, the microstructures were either fabricated individually or patterned on a per-array basis. Then, microstructures were aligned into horizontal lines under the action of an ODEP force. Figure 16A–G present the dynamic process of translating microstructures into a single structure for further biomedical and tissue applications. Furthermore, microstructures with the same shape could be assembled into the same layer, as shown in Figure 16H–L.

**Figure 17:**
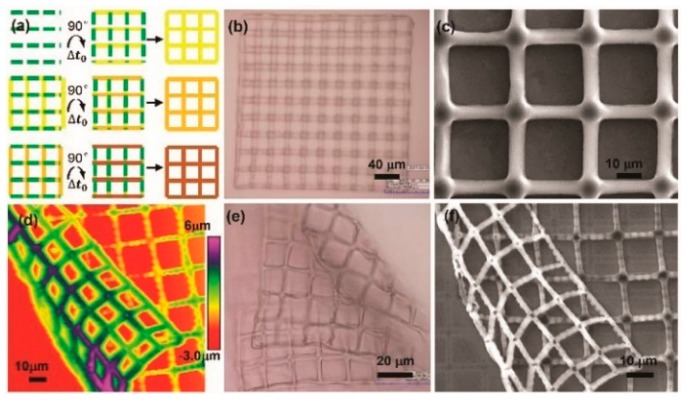
(**a**) Projected patterns alternating at a time interval (Δ*t*_0_); (**b**–**d**) are the optical microscope, SEM and AFM observation results, respectively; (**e**,**f**) are the optical microscope and SEM images of folded PEGDA hydrogel grids, respectively (reproduced from Ref. [84]).

An extended study was made on the microfabrication of 3D hydrogel scaffolds [84]. A layer-by-layer solidification mechanism was presented to fabricate hydrogel scaffolds, which involved a polymerization-delamination-polymerization loop. This loop was determined by a competition between the adhesive force and the water-absorbency-induced swelling force. The hydrogel scaffolds’ thickness ranged from tens of nanometers to hundreds of nanometers. Thin hydrogel layers were cured at the interface of the a-Si:H and PEDGA solution layers in a layer-by-layer scheme with nano-scaled thickness. Furthermore, those layers were continuously stacked along the normal direction of the OEK chip to finally construct 3D structures. Figure 17 is a microscopic characterization of the dynamic multilayered electro-polymerization of 3D hydrogel structures. Two alternating lines perpendicular to each other were designed to fabricate hydrogel microstructures, as shown in Figure 17a. The optical microscope, SEM, and AFM were employed to illustrate the fabrication results at a Δ*t*_0_ of 4 secs, as shown in Figure 17b–d, respectively. The results indicated that these mesh-like hydrogel scaffolds came with controlled pores and gaps. The folded PEGDA hydrogel grids shown in Figure 17e,f further validated the existence of pores and gaps, which could greatly facilitate the spreading, migration, and proliferation of cells as well as the mimicking of cell-cell communications.

## 6. Conclusions and Prospects

As discussed above, the OEK technique has been widely used by the microfluidic community to make a list of research achievements in terms of the manipulation and fabrication of micro/nanomaterials Although some of these achievements are also accomplished by other competing techniques, OEK is superior to the others in that it consumes ultra-low power and offers a programmable, flexible and versatile approach for parallel and multi-scaled micromanipulation. OEK has also been found to be capable of detecting cellular properties and statuses and measuring drug concentration in a label-free and dynamic manner, without relying on any other techniques [88,89,90,91,92,93,94]. These advantages suggest that OEK is well-positioned to promote the use of microfluidic tools for micro/nanomanipulation. Nonetheless, challenges remain for OEK to transform from a lab-based technique to one that is widely useful in practical applications. Here is a summary of these challenges.

Firstly, the 3D manipulation mechanism of OEK should be further explored. Early studies on the OEK technique were mostly focused on 2D manipulation schemes, which proved to be more effective than other competing techniques. With the evolvement of OEK chips, however, researchers have realized the necessity of shifting their focus to 3D manipulation. One example of this shift in focus is a two-layer a-Si:H based OEK chip, in which the top ITO glass is replaced with a-Si:H to enable spatial and adsorption-free manipulation of beads using a negative ODEP force [95]. However, this OEK chip cannot manipulate micro/nanoparticles using a positive ODEP force and requires using other OEK forces. Besides, the manipulation is liable to be affected by dynamic fluid flow and electrothermal motion, which is because the manipulated micro/nanoparticles tend to be located in the center of the chip. The poor light transmission can significantly compromise the quality of observation. A lateral-field OEK device was developed to enable parallel single-cell manipulation, which can also assemble nanowires and integrate with other microfluidic components [96]. Nevertheless, the fabrication process is complicated and involves high costs. By dynamically projecting a series of optical patterns, a layer-by-layer approach was built to create 3D microstructures in a mask-free manner [81,84]. However, these incident light patterns are only suitable for fabricating hydrogel microstructures and are insufficient for manipulating nanoparticles and assembling nanowires into functional devices. All the above problems warrant continued efforts to further enhance capabilities of OEKs in 3D manipulation.

Secondly, to facilitate the adoption of OEK-based microfluidics, it is important to develop an integrated chip that is ready for a complete and conventional laboratory-level process. This requires the integration of OEK with conventional microfluidic systems and/or components. A number of attempts have been made on such integration to move OEK further beyond its typical functions such as manipulation, separation, assembly, and fabrication of micro/nano-entities. For instance, to enhance the detection performance of OEK, a pair of optical fibers were embedded into the microchannel to rapidly and accurately count the number of particles and cells [97,98]. A lens-free holographic microscope was incorporated into the OEK chip to allow observing microparticles and cells in a large field of view [99]. This approach offers the ability to rapidly capture the holographic images of microparticles and cells across an ultra-large area of up to hundreds of square millimeters. In addition, on-chip continuous medium exchange and electroporation of cells [100] were achieved by incorporating a conventional microchannel bonded onto a top ITO glass layer. The combination of an OEK chip and surface acoustic wave elements was utilized [101,102] to provide functions such as concentration, guiding, focusing, trapping, and sorting of polystyrene beads and cell lysis. Although the abovementioned works represent tangible advances in the OEK technique, there is still a lack of effective integration of micro/nanochannels or micro/nanostructures into the liquid layer of OEK chips. This severely hinders the use of OEK in fabricating arrays of micro/nanosensors and sophisticated micro/nanodevices for bio-detection and tissue engineering applications. The fabricated micro/nanostructures mentioned in this paper still reside within the OEK chip. They cannot be delivered or directed out of the chip due to the lack of a conventional micropump or microchannel integrated into the liquid layer. Hence, there is much more to do to better integrate OEK chips with microfluidic systems or elements.

Finally, increased efforts are required to identify new possible applications of the OEK technique, which is the ultimate challenge and also the key to address the above two challenges. We believe that a technique can only flourish by bringing real benefits to end-users. Although a variety of OEK-based applications, such as separation, assembly, patterning, fabrication, and synthesis of materials, have been reported, this technique still has a long way to go to become a truly useful and practical tool in these endeavors. For example, there has not been an OEK-based method to fabricate functional micro/nanodevices for industrial applications, such as field-effect transistors and nanosensors. In sum, if upcoming studies can focus more on solving the abovementioned challenges, the OEK technique will soon be able to find wide applications in real-world situations.

## References

[1] Slota M., Keerthi A., Myers W.K., Tretyakov E., Baumgarten M., Ardavan A., Sadeghi H., Lambert C.J., Narita A., Müllen K. (2018). Magnetic edge states and coherent manipulation of graphene nanoribbons. Nature.

[2] He W., Qin C., Qiao Z., Gong Y., Zhang X., Zhang G., Chen R., Gao Y., Xiao L., Jia S. (2019). In situ manipulation of fluorescence resonance energy transfer between quantum dots and monolayer graphene oxide by laser irradiation. Nanoscale.

[3] Cui W., Mu L., Duan X., Pang W., Reed M.A. (2019). Trapping of sub-100 nm nanoparticles using gigahertz acoustofluidic tweezers for biosensing applications. Nanoscale.

[4] Khalil I., Yehye W.A., Julkapli N.M., Rahmati S., Ibn Sina A.A., Basirun W.J., Johan M.R. (2019). Graphene oxide and gold nanoparticle based dual platform with short DNA probe for the PCR free DNA biosensing using surface-enhanced Raman scattering. Biosens. Bioelectron..

[5] Kyriazi M.-E., Giust D., El-Sagheer A.H., Lackie P.M., Muskens O.L., Brown T., Kanaras A.G. (2018). Multiplexed mRNA Sensing and Combinatorial-Targeted Drug Delivery Using DNA-Gold Nanoparticle Dimers. ACS Nano.

[6] Du J., Ge H., Long S., Sun W., Fan J., Peng X. (2019). Gold nanoparticle-based plasmonic probe for selective recognition of adenosine. Sens. Actuators B Chem..

[7] Sailor M.J., Wu E.C. (2009). Photoluminescence-based sensing with porous silicon films, microparticles, and nanoparticles. Adv. Funct. Mater..

[8] Li N., Zheng J., Li C., Wang X., Ji X., He Z. (2017). An enzyme-free DNA walker that moves on the surface of functionalized magnetic microparticles and its biosensing analysis. Chem. Commun..

[9] Cauda V., Stassi S., Lamberti A., Morello M., Pirri C.F., Canavese G. (2015). Leveraging ZnO morphologies in piezoelectric composites for mechanical energy harvesting. Nano Energy.

[10] Bhattacharjee M., Pasumarthi V., Chaudhuri J., Singh A.K., Nemade H.B., Bandyopadhyay D. (2016). Self-spinning nanoparticle laden microdroplets for sensing with energy harvesting. Nanoscale.

[11] Wang S., Zhao Y., Wang M., Li H., Saqib M., Ge C., Zhang X., Jin Y. (2019). Enhancing luminol electrochemiluminescence by combined use of cobalt-based metal organic frameworks and silver nanoparticles and its application in ultrasensitive detection of cardiac troponin i. Anal. Chem..

[12] Goossens S., Navickaitė G., Monasterio C., Gupta S., Piqueras J.J., Perez R., Burwell G., Nikitskiy I., Lasanta T., Galán T. (2017). Broadband image sensor array based on graphene–CMOS integration. Nat. Photon..

[13] Li S.-X., Xia H., Xu Y.-S., Lv C., Wang G., Dai Y.-Z., Sun H.-B. (2019). Gold nanoparticle densely packed micro/nanowire-based pressure sensors for human motion monitoring and physiological signal detection. Nanoscale.

[14] Xiang N., Wang J., Li Q., Han Y., Huang D., Ni Z. (2019). Precise size-based cell separation via the coupling of inertial microfluidics and deterministic lateral displacement. Anal. Chem..

[15] Tian F., Feng Q., Chen Q., Liu C., Li T., Sun J. (2019). Manipulation of bio-micro/nanoparticles in non-newtonian microflows. Microfluid. Nanofluidics.

[16] Mao Z., Li P., Wu M., Bachman H., Mesyngier N., Guo X., Liu S., Costanzo F., Huang T.J. (2017). Enriching NanoparticlesviaAcoustofluidics. ACS Nano.

[17] Zheng T., Wang C., Xu C., Hu Q., Wei S. (2018). Patterning microparticles into a two-dimensional pattern using one column standing surface acoustic waves. Sens. Actuators A Phys..

[18] Park S., Yossifon G. (2019). Combining dielectrophoresis and concentration polarization-based preconcentration to enhance bead-based immunoassay sensitivity. Nanoscale.

[19] Liu L., Chen K., Xiang N., Ni Z. (2019). Dielectrophoretic manipulation of nanomaterials: A review. Electrophoresis.

[20] Zhang Y., DaSilva M., Ashall B., Doyle G., Zerulla D., Sands T.D., Lee G.U. (2011). Magnetic manipulation and optical imaging of an active plasmonic single-particle Fe–Au nanorod. Langmuir.

[21] Ebrahimian H., Giesguth M., Dietz K.-J., Reiss G., Herth S. (2014). Magnetic tweezers for manipulation of magnetic particles in single cells. Appl. Phys. Lett..

[22] Nan F., Yan Z. (2019). Silver-nanowire-based interferometric optical tweezers for enhanced optical trapping and binding of nanoparticles. Adv. Funct. Mater..

[23] Gould O.E.C., Box S.J., Boott C.E., Ward A.D., Winnik M.A., Miles M.J., Manners I. (2019). Manipulation and deposition of complex, functional block copolymer nanostructures using optical tweezers. ACS Nano.

[24] Manna R.K., Shklyaev O.E., Kauffman J., Tansi B., Sen A., Balazs A.C. (2019). Light-induced convective segregation of different sized microparticles. ACS Appl. Mater. Interfaces.

[25] Tsuji T., Matsumoto Y., Kugimiya R., Doi K., Kawano S. (2019). Separation of nano—and microparticle flows using thermophoresis in branched microfluidic channels. Micromachines.

[26] Li H., Han Y., Duan T., Leifer K. (2019). Size-dependent elasticity of gold nanoparticle measured by atomic force microscope based nanoindentation. Appl. Phys. Lett..

[27] Moreno M.M., Ares P., Moreno C., Zamora F., Gomez-Navarro C., Herrero J.G. (2019). AFM manipulation of gold nanowires to build electrical circuits. Nano Lett..

[28] Ahmed D., Baasch T., Blondel N., Läubli N., Dual J., Nelson B.J. (2017). Neutrophil-inspired propulsion in a combined acoustic and magnetic field. Nat. Commun..

[29] Wu M., Ouyang Y., Wang Z., Zhang R., Huang P.-H., Chen C., Li H., Li P., Quinn D., Dao M. (2017). Isolation of exosomes from whole blood by integrating acoustics and microfluidics. Proc. Natl. Acad. Sci. USA.

[30] Zhao W., Yu H., Wen Y., Wang F., Yang Y., Liu Z., Liu L., Li W.J. (2019). Detection of micro/nano-particle concentration using modulated light-emitting diode white light source. Sens. Actuators A Phys..

[31] Shkolnikov V., Xin D., Chen C. (2019). Continuous dielectrophoretic particle separation via isomotive dielectrophoresis with bifurcating stagnation flow. Electrophoresis.

[32] Chiou P.Y., Ohta A.T., Wu M.C. (2005). Massively parallel manipulation of single cells and microparticles using optical images. Nature.

[33] Yang X., Niu X., Liu Z., Zhao Y., Zhang G., Liang W., Li W.J. (2017). Accurate extraction of the self-rotational speed for cells in an electrokinetics force field by an image matching algorithm. Micromachines.

[34] Liang W., Wang Y., Zhang H., Liu L. (2016). Characterization of the self-rotational motion of stored red blood cells by using optically-induced electrokinetics. Opt. Lett..

[35] Liang W., Zhang K., Yang X., Liu L., Yu H., Zhang W. (2015). Distinctive translational and self-rotational motion of lymphoma cells in an optically induced non-rotational alternating current electric field. Biomicrofluidics.

[36] Liang W., Zhao Y., Liu L., Wang Y., Dong Z., Li W.J., Lee G.-B., Xiao X., Zhang W. (2014). Rapid and Label-Free Separation of Burkitt’s Lymphoma Cells from Red Blood Cells by Optically-Induced Electrokinetics. PLoS ONE.

[37] Ke L.-Y., Kuo Z.-K., Chen Y.-S., Yeh T.-Y., Dong M., Tseng H.-W., Liu C.-H. (2018). Cancer immunotherapy μ-environment LabChip: taking advantage of optoelectronic tweezers. Lab Chip.

[38] Kamata M., Taguchi Y., Nagasaka Y. (2018). Design of an optofluidic diffusion sensor by transient grating using dielectrophoresis. Opt. Express.

[39] Wu M.C. (2011). Optoelectronic tweezers. Nat. Photon..

[40] Hwang H., Park J.-K. (2011). Optoelectrofluidic platforms for chemistry and biology. Lab Chip.

[41] Liang W., Liu L., Zhang H., Wang Y., Li W.J. (2019). Optoelectrokinetics-based microfluidic platform for bioapplications: A review of recent advances. Biomicrofluidics.

[42] Liang W., Liu N., Dong Z., Liu L., Mai J.D., Lee G.-B., Li W.J. (2013). Simultaneous separation and concentration of micro- and nano-particles by optically induced electrokinetics. Sens. Actuators A Phys..

[43] Castellanos A., Ramos A., González A., Green N.G., Morgan H. (2003). Electrohydrodynamics and dielectrophoresis in microsystems: scaling laws. J. Phys. D Appl. Phys..

[44] Valley J.K., Jamshidi A., Ohta A.T., Hsu H.-Y., Wu M.C. (2008). Operational regimes and physics present in optoelectronic tweezers. J. Microelectrom. Syst..

[45] Chiou P.-Y., Ohta A., Jamshidi A., Hsu H.-Y., Wu M. (2008). Light-actuated AC electroosmosis for nanoparticle manipulation. J. Microelectrom. Syst..

[46] Hwang H., Park J.-K. (2009). Rapid and selective concentration of microparticles in an optoelectrofluidic platform. Lab Chip.

[47] Wang W., Lin Y.-H., Guan R.-S., Wen T.-C., Guo T.-F., Lee G.-B. (2009). Bulk-heterojunction polymers in optically-induced dielectrophoretic devices for the manipulation of microparticles. Opt. Express.

[48] Wang W., Lin Y.-H., Wen T.-C., Guo T.-F., Lee G.-B. (2010). Selective manipulation of microparticles using polymer-based optically induced dielectrophoretic devices. Appl. Phys. Lett..

[49] Lin S.-J., Hung S.-H., Jeng J.-Y., Guo T.-F., Lee G.-B. (2012). Manipulation of micro-particles by flexible polymer-based optically-induced dielectrophoretic devices. Opt. Express.

[50] Yang S.-M., Yu T.-M., Huang H.-P., Ku M.-Y., Hsu L., Liu C.-H. (2010). Dynamic manipulation and patterning of microparticles and cells by using TiOPc-based optoelectronic dielectrophoresis. Opt. Lett..

[51] Yang S.-M., Yu T.-M., Huang H.-P., Ku M.-Y., Tseng S.-Y., Tsai C.-L., Chen H.-P., Hsu L., Liu C.-H. (2011). Light-driven manipulation of picobubbles on a titanium oxide phthalocyanine-based optoelectronic chip. Appl. Phys. Lett..

[52] Yu T.-M., Yang S.-M., Fu C.-Y., Liu M.-H., Hsu L., Chang H.-Y., Liu C.-H. (2013). Integration of organic opto-electrowetting and poly(ethylene) glycol diacrylate (PEGDA) microfluidics for droplets manipulation. Sens. Actuators B Chem..

[53] Yang S.-M., Tseng S.-Y., Chen H.-P., Hsu L., Liu C.-H. (2013). Cell patterning via diffraction-induced optoelectronic dielectrophoresis force on an organic photoconductive chip. Lab Chip.

[54] Hwang H., Park Y.-H., Park J.-K. (2009). Optoelectrofluidic control of colloidal assembly in an optically induced electric field. Langmuir.

[55] Huang J.-Y., Lu Y.-S., Yeh J.A. (2006). Self-assembled high NA microlens arrays using global dielectricphoretic energy wells. Opt. Express.

[56] Lin W.-Y., Lin Y.-H., Lee G.-B. (2010). Separation of micro-particles utilizing spatial difference of optically induced dielectrophoretic forces. Microfluid. Nanofluidics.

[57] Park S.-Y., Kalim S., Callahan C., Teitell M.A., Chiou E.P.Y. (2009). A light-induced dielectrophoretic droplet manipulation platform. Lab Chip.

[58] Lee H., Hwang H., Park J.-K. (2009). Generation and manipulation of droplets in an optoelectrofluidic device integrated with microfluidic channels. Appl. Phys. Lett..

[59] Hung S., Lin Y., Lee G. (2010). A microfluidic platform for manipulation and separation of oil-in-water emulsion dielectrophoresis. J Micromechan. Microeng..

[60] Zhang S., Juvert J., Cooper J.M., Neale S.L. (2016). Manipulating and assembling metallic beads with optoelectronic tweezers. Sci. Rep..

[61] Liang W., Wang S., Dong Z., Lee G.-B., Li W.J. (2012). Optical spectrum and electric field waveform dependent optically-induced dielectrophoretic (ODEP) micro-manipulation. Micromachines.

[62] Han D., Hwang H., Park J.-K. (2013). Optoelectrofluidic behavior of metal–polymer hybrid colloidal particles. Appl. Phys. Lett..

[63] Zhang S., Scott E.Y., Singh J., Chen Y., Zhang Y., Elsayed M., Chamberlain M.D., Shakiba N., Adams K., Yu S. (2019). The optoelectronic microrobot: A versatile toolbox for micromanipulation. Proc. Natl. Acad. Sci. USA.

[64] Jamshidi A., Pauzauskie P.J., Schuck P.J., Ohta A.T., Chiou P.-Y., Chou J., Yang P., Wu M.C. (2008). Dynamic manipulation and separation of individual semiconducting and metallic nanowires. Nat. Photon..

[65] Pauzauskie P.J., Jamshidi A., Valley J.K., Satcher J.H., Wu M.C. (2009). Parallel trapping of multiwalled carbon nanotubes with optoelectronic tweezers. Appl. Phys. Lett..

[66] Lee M.-W., Lin Y.-H., Lee G.-B. (2010). Manipulation and patterning of carbon nanotubes utilizing optically induced dielectrophoretic forces. Microfluid. Nanofluidics.

[67] Lin Y.-H., Ho K.-S., Yang C.-T., Wang J.-H., Lai C.-S. (2014). A highly flexible platform for nanowire sensor assembly using a combination of optically induced and conventional dielectrophoresis. Opt. Express.

[68] Lim M.B., Felsted R.G., Zhou X., Smith B.E., Pauzauskie P.J. (2018). Patterning of graphene oxide with optoelectronic tweezers. Appl. Phys. Lett..

[69] Jamshidi A., Neale S.L., Yu K., Pauzauskie P.J., Schuck P.J., Valley J.K., Hsu H.-Y., Ohta A.T., Wu M.C. (2009). NanoPen: Dynamic, Low-power, and light-actuated patterning of nanoparticles. Nano Lett..

[70] Yang S.-M., Harishchandra P.T., Yu T.-M., Liu M.-H., Hsu L., Liu C.-H. (2011). Concentration of magnetic beads utilizing light-induced electro-osmosis flow. IEEE Trans. Magn..

[71] Ota S., Wang S., Wang Y., Yin X., Zhang X. (2013). Lipid bilayer-integrated optoelectronic tweezers for nanoparticle manipulations. Nano Lett..

[72] Liang W., Liu L., Lai S.H.-S., Wang Y., Lee G.-B., Li W.J. (2014). Rapid assembly of gold nanoparticle-based microstructures using optically-induced electrokinetics. Opt. Mater. Express.

[73] Liu N., Liang W., Mai J.D., Liu L., Lee G.-B., Li W.J. (2014). Rapid fabrication of nanomaterial electrodes using digitally controlled electrokinetics. IEEE Trans. Nanotechnol..

[74] Liang W., Liu L., Wang Y., Lee G.-B., Li W.J. (2018). Rapid assembly of CARBON Nanoparticles into electrical elements by optically-induced electroosmotic flow. IEEE Trans. Nanotechnol..

[75] Liu N., Wei F., Liu L., Lai H.S.S., Yu H., Wang Y., Lee G.-B., Li W.J. (2015). Optically-controlled digital electrodeposition of thin-film metals for fabrication of nano-devices. Opt. Mater. Express.

[76] Liu N., Wang F., Liu L., Yu H., Xie S., Wang J., Wang Y., Lee G.-B., Li W.J. (2016). Rapidly patterning micro/nano devices by directly assembling ions and nanomaterials. Sci. Rep..

[77] Li P., Liu N., Yu H., Wang F., Liu L., Lee G.-B., Wang Y., Li W.J. (2016). Silver nanostructures synthesis via optically induced electrochemical deposition. Sci. Rep..

[78] Li M., Liu N., Li P., Shi J., Li G., Xi N., Wang Y., Liu L. (2017). Performance investigation of multilayer MoS2 thin-film transistors fabricated via mask-free optically induced electrodeposition. ACS Appl. Mater. Interfaces.

[79] Liu N., Li M., Liu L., Yang Y., Mai J., Pu H., Sun Y., Li W.J. (2018). Single-step fabrication of electrodes with controlled nanostructured surface roughness using optically-induced electrodeposition. J. Micromechan. Microeng..

[80] Wang S., Liang W., Dong Z., Lee V.G.B., Li W.J. (2011). Fabrication of micrometer—and nanometer-scale polymer structures by visible light induced dielectrophoresis (DEP) force. Micromachines.

[81] Liu N., Li P., Liu L., Yu H., Wang Y., Lee G.-B., Li W.J. (2015). 3-D Non-UV digital printing of hydrogel microstructures by optically controlled digital electropolymerization. J. Microelectrom. Syst..

[82] Li Y., Lai S.H.S., Liu N., Zhang G., Liu L., Lee G.-B., Li W.J. (2016). Fabrication of high-aspect-ratio 3D Hydrogel microstructures using optically induced electrokinetics. Micromachines.

[83] Yang W., Yu H., Li G., Wang Y., Liu L. (2017). Tissue engineering: High-throughput fabrication and modular assembly of 3D heterogeneous microscale tissues (small 5/2017). Small.

[84] Li P., Yu H., Liu N., Wang F., Lee G.-B., Wang Y., Liu L., Li W.J. (2018). Visible light induced electropolymerization of suspended hydrogel bioscaffolds in a microfluidic chip. Biomater. Sci..

[85] Liu N., Liang W., Liu L., Wang Y., Mai J.D., Lee G.-B., Li W.J. (2014). Extracellular-controlled breast cancer cell formation and growth using non-UV patterned hydrogels via optically-induced electrokinetics. Lab Chip.

[86] Chan-Park M.B., Yan Y., Neo W.K., Zhou W., Zhang J., Yue C.Y. (2003). Fabrication of high aspect ratio poly(ethylene glycol)-containing microstructures by UV embossing. Langmuir.

[87] Terakawa M., Torres-Mapa M.L., Takami A., Heinemann D., Nedyalkov N.N., Nakajima Y., Hördt A., Ripken T., Heisterkamp A. (2016). Femtosecond laser direct writing of metal microstructure in a stretchable poly(ethylene glycol) diacrylate (PEGDA) hydrogel. Opt. Lett..

[88] Zhao Y., Liang W., Zhang G., Mai J.D., Liu L., Lee G.-B., Li W.J. (2013). Distinguishing cells by their first-order transient motion response under an optically induced dielectrophoretic force field. Appl. Phys. Lett..

[89] Liang W., Zhao Y., Liu L., Wang Y., Li W.J., Lee G.-B. (2017). Determination of cell membrane capacitance and conductance via optically induced electrokinetics. Biophys. J..

[90] Zhao Y., Lai H.S.S., Zhang G., Lee G.-B., Li W.J. (2015). Measurement of single leukemia cell’s density and mass using optically induced electric field in a microfluidics chip. Biomicrofluidics.

[91] Chiu T.-K., Chao A.-C., Chou W.-P., Liao C.-J., Wang H.-M., Chang J.-H., Chen P.-H., Wu M.-H. (2018). Optically-induced-dielectrophoresis (ODEP)-based cell manipulation in a microfluidic system for high-purity isolation of integral circulating tumor cell (CTC) clusters based on their size characteristics. Sens. Actuators B Chem..

[92] Chu P.-Y., Liao C.-J., Hsieh C.-H., Wang H.-M., Chou W.-P., Chen P.-H., Wu M.-H. (2019). Utilization of optically induced dielectrophoresis in a microfluidic system for sorting and isolation of cells with varied degree of viability: Demonstration of the sorting and isolation of drug-treated cancer cells with various degrees of anti-cancer drug resistance gene expression. Sens. Actuators B Chem..

[93] Huang S.-H., Hung L.-Y., Lee G.-B. (2016). Continuous nucleus extraction by optically-induced cell lysis on a batch-type microfluidic platform. Lab Chip.

[94] Hsiao Y.-C., Wang C.-H., Lee W.-B., Lee G.-B. (2018). Automatic cell fusion via optically-induced dielectrophoresis and optically-induced locally-enhanced electric field on a microfluidic chip. Biomicrofluidics.

[95] Hwang H., Oh Y., Kim J.-J., Choi W., Park J.-K., Kim S.-H., Jang J. (2008). Reduction of nonspecific surface-particle interactions in optoelectronic tweezers. Appl. Phys. Lett..

[96] Valley J.K., Ohta A.T., Neale S.L., Jamshidi A., Wu M.C., Hsu H.-Y. (2009). Optoelectronic tweezers as a tool for parallel single-cell manipulation and stimulation. IEEE Trans. Biomed. Circuits Syst..

[97] Lin Y.-H., Lee G.-B. (2008). Optically induced flow cytometry for continuous microparticle counting and sorting. Biosens. Bioelectron..

[98] Lin Y.-H., Lee G.-B. (2010). An integrated cell counting and continuous cell lysis device using an optically induced electric field. Sens. Actuators B Chem..

[99] Huang K.-W., Su T.-W., Ozcan A., Chiou P.-Y. (2013). Optoelectronic tweezers integrated with lensfree holographic microscopy for wide-field interactive cell and particle manipulation on a chip. Lab Chip.

[100] Lee G.-B., Chang C.-J., Wang C.-H., Lu M.-Y., Luo Y.-Y. (2015). Continuous medium exchange and optically induced electroporation of cells in an integrated microfluidic system. Microsyst. Nanoeng..

[101] Witte C., Kremer C., Cooper J.M., Neale S.L. (2013). Continuous cell lysis in microfluidics through acoustic and optoelectronic tweezers. SPIE MOEMS-MEMS.

[102] Witte C., Wilson R., Cooper J.M., Neale S.L. OET Meets Acoustic Tweezing. Proceedings of the Optical Trapping and Optical Micromanipulation IX.

